# Effects of Blood Pressure on Cognitive Performance: A Systematic Review

**DOI:** 10.3390/jcm9010034

**Published:** 2019-12-22

**Authors:** Giuseppe Forte, Vilfredo De Pascalis, Francesca Favieri, Maria Casagrande

**Affiliations:** 1 Dipartimento di Psicologia, Università di Roma “Sapienza”, 00185 Roma, Italy; vilfredo.depascalis@uniroma1.it (V.D.P.); francesca.favieri@uniroma1.it (F.F.); 2 Dipartimento di Psicologia Dinamica e Clinica, Università di Roma “Sapienza”, 00185 Roma, Italy

**Keywords:** blood pressure, cognitive functions, attention, executive functions, language, processing speed, memory, visuospatial abilities, cognitive impairment

## Abstract

Background: High blood pressure has been associated with an increased risk of developing cognitive impairment. However, this relationship is unclear. This study aims to systematically review the effects of blood pressure on executive functioning, language, memory, attention and processing speed. Methods: The review process was conducted according to the PRISMA-Statement, using the PubMed, PsycINFO, PsycARTICLES and MEDLINE databases. Restrictions were made by selecting studies, which included one or more cognitive measures and reported blood pressure recordings. Studies that included participants with medical conditions or people diagnosed with dementia, psychiatric disorders, stroke and head trauma were excluded. The review allows selecting fifty studies that included 107,405 participants. The results were reported considering different cognitive domains separately: global cognitive functioning, attention, processing speed, executive functions, memory and visuospatial abilities. Results: Higher blood pressure appears to influence cognitive performance in different domains in the absence of dementia and severe cardiovascular diseases, such as strokes. This relationship seems to be independent of demographic factors (gender and education), medical co-morbidity (diabetes), and psychiatric disorders (depression). Furthermore, it presents different patterns considering ageing. In the elderly, a sort of “cardiovascular paradox” is highlighted, which allows considering higher blood pressure as a protective factor for cognitive functioning. Conclusions: The results underline that higher blood pressure is associated with a higher risk of cognitive decline in people without dementia or stroke. These findings highlight the need to introduce early management of blood pressure, even in the absence of clinical hypertension, to prevent the risk of a decline of cognitive functioning typically associated with ageing.

## 1. Introduction

During the ageing process, several factors can contribute to the physiological decline of global cognitive functions or specific cognitive domains [1,2]. The cognitive decline can be due to both non-modifiable factors (hormonal changes, genetic factors, trauma, etc.) and modifiable factors (lifestyles, prolonged stress, etc.) (e.g., [3]), and it is usually manifested by mnestic difficulties [4], poor mental flexibility [5] and lower ability to inhibit responses [6]. Several authors tried to investigate the causes of this physiological, cognitive decline, highlighting that ageing is characterised by volumetric reduction of grey and white matter [7], decrease of dopamine levels [8], presence of oxidative stress [9], and cardiovascular dysfunctions of both the arterial blood pressure [10] and the heart rate variability [11,12]. The severity of cognitive decline and the rapidity with which it occurs are unique to each individual. In some cases, cognitive difficulties become clinically relevant, evolving into dementia. Given the progressive ageing of the world population, the challenge of modern science is highlighted the factors associated with the evolution of cognitive impairment.

Cognitive impairment appears to be amplified by several factors as the habit of smoking cigarettes [13,14], chronic use of alcohol [15], poor eating habits and obesity [16,17], inadequate exercise [18], chronic stress [19] or by some pathologies such as diabetes mellitus [20] or depression [21]. Moreover, the risk of cognitive impairment appears to increase in the presence of cardiovascular disorders, such as high blood pressure, cardiomyopathies, arteriosclerosis, cerebral infarcts and strokes [22]. Many studies have shown an association between vascular ageing indices, such as stiffness of the arteries and dysfunction of small vessels and cognitive decline [23]. The exposure to high blood pressure would damage the cerebral microcirculation, causing cognitive impairments [23].

The mechanisms that can explain the relationship between high blood pressure and cognitive impairment are not wholly known. Some studies have shown associations between high blood pressure and white matter lesions, lacunar infarcts, reduced cerebral volume and cerebral haemorrhages [24]. High blood pressure affects cerebral perfusion, causing adaptive vascular changes, and it also accelerates arteriosclerotic changes in the brain and predisposes to a narrowing of the vessels and an increase in resistance [25]. These changes cause alterations in the physiological processes of cerebral blood flow regulation, making hypertensive patients more vulnerable to episodes of hypotension that may play a central role in the development of cerebrovascular damage [26].

Some studies (e.g., [27]) have shown that alterations of cerebral perfusion due to hypertension can alter neural functioning and metabolism, with consequent changes in the white matter. Furthermore, exposure to high blood pressure contributes to an accumulation of beta-amyloid in the brain [28]. However, these structural brain changes can also be related to aspect other than vascular and blood pressure alteration, as reported by studies which analysed the aetiology of dementia disorders as Alzheimer Disease [29,30], focusing their attention on molecular or genetic causes of these disorders.

The increase in arterial pressure is also associated with an adrenergic hyperactivation and a simultaneous reduction of the vagal tone [31,32]. These autonomic dysfunctions may precede and contribute to both the maintenance and progression of hypertension [33] and are related to cognitive dysfunctions [12]. This pathophysiological process could explain the association between hypertension and the deterioration of brain functions, specifically of executive functions [33,34], language [35], attention [36], processing speed and memory [37].

This systematic review of the literature analysed whether high blood pressure represents a risk factor for the decline of different cognitive domains. Moreover, it points to evaluate whether some cognitive functions, more than others, are negatively affected by high blood pressure.

## 2. Method

The review process was conducted according to the PRISMA-Statement [38,39]. The protocol has not been registered.

### 2.1. Research Strategies

A systematic analysis of international literature was conducted, selecting articles published in peer-review journals by using PubMed, PsycINFO, PsycARTICLES and MEDLINE databases. The last research was done on 10 June 2018. Restrictions were made, limiting the research to academic publications with full-text in English, studies on humans’ populations, without restrictions respect to age, gender and ethnicity. The search strategy used the following keywords: “cognitive function*”; “cognitive impairment”; “blood pressure”; “hypertension”; “high blood pressure”. Table 1 presented the syntax used in the search.

### 2.2. Eligibility Criteria

The list of potential articles produced by systematic research has been revised. Studies that included one or more cognitive processes and reported the measurement of blood pressure were selected. Studies that included participants with medical conditions that could potentially influence the investigated relationship and those that included participants diagnosed with dementia, psychiatric disorders, strokes, and head traumas were excluded.

The reading of title and abstract allowed the first exclusion of not inherent studies. A further selection was made by reading the full-text. Studies that presented methodological criticisms or did not report essential data were excluded. Two researchers made the collection independently. Any problems have been resolved through a supervisor.

### 2.3. Data Collection and Quality Assessment

According to the PICOS approach [38], information on the author(s), year of publication, number and characteristics of the participants (age, years of education, gender, mean systolic blood pressure, mean diastolic blood pressure); cognitive domains analysed; type and direction of the identified relationship; possible presence of follow-up have been extracted by each study. Table 2 reports the selected data.

According to the definition provided by the authors, the neuropsychological tests used in the studies were associated to some cognitive domains determined a priori (global cognitive functions, attention, memory, executive functions, visuospatial abilities, processing speed). The performance in each domain was analysed, considering a single test or a composite score based on multiple neuropsychological tests used for the assessment (see Table 3).

The methodological quality of the studies was assessed using the criteria from the Cochrane Handbook for Systematic Review [40], modified ad hoc according to the aims of this systematic review. The quality of different studies was categorised as unclear/low/high risk of bias (“0” for a low risk of bias, “1” for a medium risk of bias, “2” for a high risk of bias, “Unclear” otherwise). For each study was calculated the mean score, and it was multiplied by 100. Then, studies were categorised in low risk of bias (lower than 75%) or high risk of bias (higher than 75%). Finally, if at least two items were unclear, the studies were classified as unclear risk of bias. The items included: the use of international guidelines for the assessment and measurement of blood pressure (selection bias), the selection of sample and the control of confounding variables (selection bias), the use of appropriate tasks for the analyses of the cognitive domains considered (detection bias), incomplete outcome data (attrition bias), selective reporting (reporting bias) and other biases. 

## 3. Results

### 3.1. Studies Selection

The initial search produced 9335 articles. After excluding 1842 duplicates, 7443 articles were rejected according to the analysis of both title and abstract. Finally, 277 studies were reviewed and subjected to the quality assessment. At the end of the review process, 50 articles were identified as fitted with the aims of this systematic review.

**Figure 1:**
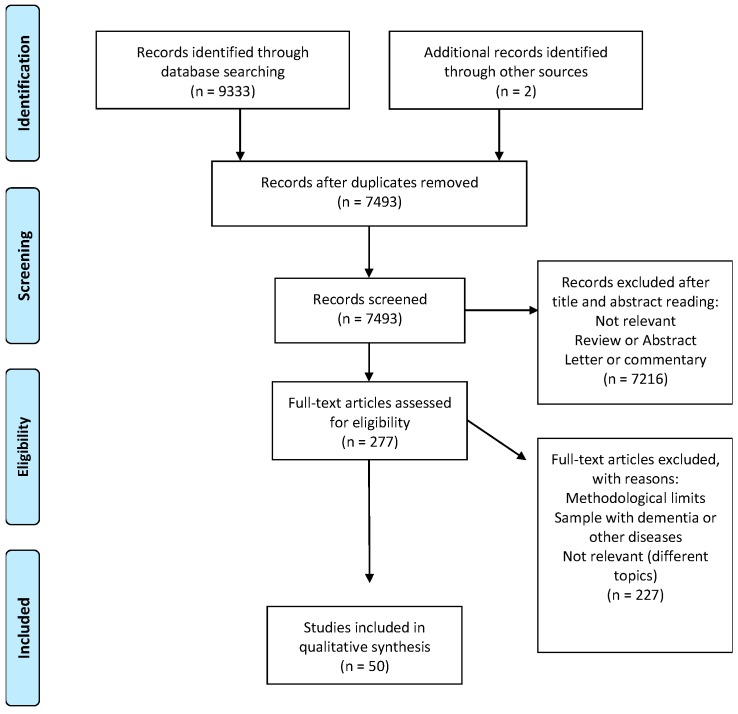
PRISMA flow chart of the selection process.

**Figure 2:**
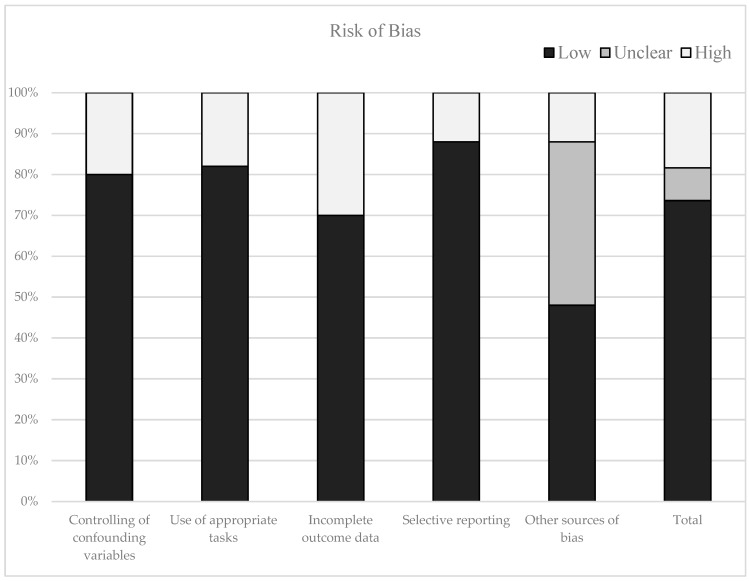
Risk of bias in the selected studies.

The flow chart (Figure 1) shows the number of studies identified by the bibliographic databases and the number of studies examined, assessed for eligibility and included in the review with the reasons of exclusion. A summary of the quality assessment is presented in Figure 2.

### 3.2. Quality Assessment

Table 2 shows the percentage of articles fulfilling each quality criterion assessed. On average, the quality of the studies was good (88%), 44 studies presented low scores on the risk of bias. Six studies (11%) showed high scores. A large percentage of the studies used valid and reliable tools for measuring cognitive performance and included an appropriate sample size. Moreover, most researches adequately controlled for confounding variables. The higher risk of bias is due to incomplete outcome data (see Figure 2).

### 3.3. Demographic Features

The fifty studies that met the inclusion criteria were conducted from 1990 to 2018 and involved 107,405 people. Participants were aged between 18 [76] and 100 years [89]. The studies showed a variable percentage of men between 17 [53] and 69 [67]. In some cases, samples size were composed only by women [72] and in other cases, there were samples of only men [43,47,65,80,83,84]. Four studies have made a gender comparison [56,63,71,85]. However, in some studies, we did not find information about gender composition (see Table 2). Seventeen studies performed a longitudinal analysis (see Table 2). These studies included follow-ups ranging from 3 [52,86] to 30 years [48].

Given the multidimensionality of the constructs examined, many studies included some confounding variables (gender, age, ethnicity, etc.) in the statistical analyses.

### 3.4. Blood Pressure Measurements

All the studies which were included in this review assessed blood pressure using an indirect measurement of the brachial artery with a sphygmomanometer, taking into account both systolic and diastolic blood pressure.

Eleven studies [75,80,81,82,83,85,86,87,88,89,90] made a comparison between hypertensive and normotensive individuals. According to the international guidelines [91,92]. People were considered as hypertensive when presented an arterial blood pressure higher than 140/90 mmHg.

### 3.5. Cognitive Domains

Twenty-nine studies have investigated global cognitive functions, thirty-five considered memory, ten analysed language, thirteen evaluated attention, twenty-nine assessed executive functions, nineteen examined processing speed and nineteen measured visuospatial abilities (for references see Table 2).

### 3.6. Blood Pressure and Global Cognitive Functioning

Global cognitive functioning appears to be influenced by blood pressure. However, in some cases, studies showed inconsistent results. On the one hand, high blood pressure was associated with worse cognitive performance [41,67,68,72,73,78]. A poor performance was also associated with higher systolic blood pressure [43,48,62,66,69], or with higher diastolic blood pressure only [44,47]. On the other hand, excessively low blood pressure was related to a decline of global cognitive performance [55,58,65,77]. Some studies reported that cognitive performance was associated with blood pressure by a J-shaped [49,52] or U-shaped [54] curvilinear relationship.

The control of possible confounding variables allowed highlighting the influence of blood pressure on cognitive impairment only in older people [42,79] or in males [56].

The presence of a relationship between high blood pressure and cognitive impairment appears to be confirmed by studies comparing hypertensives and normotensives. In these studies, people with hypertension showed a higher decline of global cognitive functioning [72,75,88] than normotensive.

In contrast to these findings which confirmed a role of high blood pressure on global cognitive impairment, few studies did not report any relationship, considering both a healthy population [46,50] and hypertensive patients [89].

### 3.7. Blood Pressure and Memory

Memory abilities appear to be associated with both high [41,48,51,53,57,63,67,71,72,73,75,76,81,84] and low [55,65] blood pressure. This relationship appeared stronger with increasing age [42,79,80] and in men [45,56], and was also confirmed in hypertensive patients [61,83,85,88].

Some studies have reported J-shaped [49] or U-shaped [54,61] curvilinear relationships between blood pressure and mnestic performance. However, some studies did not confirm any relationship between blood pressure and memory [59,70,78,86,90].

### 3.8. Blood Pressure and Language

Elevated diastolic blood pressure [57] or high general blood pressure [63,73,82,86] were associated with poor linguistic performance. However, two studies [65,70] showed that poor linguistic performance was related to low blood pressure. The results of some studies showed that age could mediate this relationship [42] and that the association between linguistic performance and blood pressure is non-linear [46,54]. Finally, two studies did not show any relationship between linguistic performance and blood pressure [78,83].

### 3.9. Blood Pressure and Attention

The studies showed that high overall blood pressure [64,73,78,81] or hypertension [85,88] negatively affect attentional performance. However, in some cases, this relationship was age-mediated [42,80] or followed a U-shaped curve [61].

Conversely, some studies identified a positive effect of high blood pressure on attentive performance [64,70], while others did not highlight any relationship [53,56,61].

### 3.10. Blood Pressure and Executive Functions

High blood pressure, considering systolic blood pressure [59,62], diastolic blood pressure [47,53], or both [41,51,63,67,72,73,75,76,78,82,83,84] appears to be associated with an executive impairment. Only one study [65] showed that high blood pressure affected positively executive functioning.

The relationship between blood pressure and executive functioning could be mediated by age [42,80] or gender [45], highlighting how this relationship appears strengthened with increasing age and in men.

When studies considered hypertensive patients, they found that this condition was associated with a lower executive performance [86,87,88,90]. However, the relationship between hypertension and executive functioning was not confirmed by all the studies [48,56,85].

### 3.11. Blood Pressure and Processing Speed

Blood pressure negatively affects information processing speed. A higher general blood pressure [72,73,76,88,90] or an elevated systolic blood pressure [48,62,71] are associated with slower information processing. However, one study showed a positive relationship between low blood pressure and poor performance in processing speed [64]. Some studies observed a non-linear relationship [46] or a U-shaped relationship [54,61]. The selected researches also showed gender and age as possible mediators, highlighting that the relationship between blood pressure and performance in processing speed was stronger in men [45] and older people [80].

This relationship was confirmed, also considering hypertensive patients [83,85,87]. However, one study did not confirm these results [82].

### 3.12. Blood Pressure and Visuospatial Abilities

Visuospatial abilities seem impaired in people with high systolic blood pressure [48], high diastolic blood pressure [47,53,57], or with elevated values of both diastolic and systolic blood pressure [41,83]. This trend was confirmed even considering hypertensive patients, who exhibited worse performance than normotensives individuals [65,66,67,75,81,85]. In some cases, higher impairment was observed with increasing age [86] and in men [56,80]. Some studies reported a non-linear relationship between blood pressure and visuospatial performance [42,61]. Only one study found a positive association between blood pressure and visuospatial performance [64].

Finally, some authors [59,61,70,82] did not show any influence of blood pressure on visuospatial functions.

## 4. Discussion

This review was aimed to analyse the presence and the nature of the relationship between blood pressure and cognitive functions. A large number of studies that satisfied the inclusion criteria confirmed the high interest of the research on this topic. This interest is due to the health implications. High blood pressure is an indirect cause of 7.5 million deaths (12% of total deaths) [91], and it is independently and linearly correlated with an increased risk of developing cardio and cerebrovascular diseases [92,93]. These complications are often associated with severe cognitive impairment [94], increasing risk of developing dementia, or other vascular and non-vascular pathologies (for a review, see [95]). All these aspects underline the importance to analyse better the role of the exposure to high blood pressure on cognitive impairment.

The qualitative analysis of the selected studies highlighted the presence of low risk of bias in the selected studies, the employments of valid and reliable tools for measuring cognitive performance, the inclusion of appropriate sample size and an adequate control for confounding variables. All these aspects make the selected studies reliable and their results generalizable.

The studies which compared hypertensive and normotensive people reported robust results.

Long-term hypertension appears to be led to alteration of the cerebral flow and, therefore, to higher impairment of the cognitive performance, making the worsening of cognitive functioning more pronounced. The analyses of the effects of high blood pressure in the general population reported more inconsistent results, despite giving useful information from a preventive perspective.

Studies comparing hypertensive with normotensive showed an association between hypertension condition (quantified as blood pressure equal to or higher than 140/90 mmHg) and cognitive impairment, considering both global cognitive functioning and specific cognitive domains.

However, the spectrum of the cognitive domain is not exhaustively investigated. The higher interest for specific cognitive domains, such as memory and executive functions, did not allow robust conclusions for all the cognitive functions.

Not all the studies confirmed the relationship between blood pressure and cognitive domains (e.g., attention [53,56]; memory [73,86]; Executive functions [48,61]; Processing Speed and Visuospatial Skills [82]). These negative findings could be due to heterogeneous and small samples or to the characteristics of the tasks used to assess the performance.

The results on attention are particularly interesting because, unexpectedly, some studies indicated that high blood pressure could be linked to better performance [64,70]. These results could be due to increased cerebral perfusion that would be responsible for better attentional performance [10]. However, the reasons why higher cerebral perfusion should improve attentional functions but not the other cognitive domains are not clear. This finding should be validated in further studies considering the use of tasks that, unlike those used in the selected studies, permit a complete identification of the construct (i.e., Attentional Network Task or its variants [96,97,98,99,100]).

Another interesting result is that reported by Huang and colleagues [89]. The authors, considering an old–old population (ninety years old and centenarians), showed that in old age, hypertension does not seem to have a role in the decline of cognitive functions. However, the specificity of the sample considered, the unbalanced gender, and the natural selection of participants do not lead to generalising these findings.

Although the results of the studies on blood pressure agree with those found by considering hypertension, the relationship between blood pressure and cognitive functions is not always linear. Some studies [54,60,61] showed a curvilinear relationship indicating that both high and low blood pressure may be related to cognitive impairment. These results are in line with the findings reported by neuroimaging studies observing that both excessive and low blood flow can be dangerous for optimal brain functioning [101,102]. On the one hand, high blood pressure can cause strokes and lesions of the white matter. On the other, the effect of low arterial pressure appears to be associated with ischemic lesions [54]. Therefore, it would be necessary to maintain homeostatic blood pressure to preserve cognitive functioning.

An interesting aspect that emerges from this review is the role of some variables, such as gender and age, in modulating the relationship between blood pressure and cognitive functioning. About the gender, some studies observed that higher blood pressure is related to cognitive impairment in women [45], while other authors find opposite results, highlighting a higher impairment in men [56,63]. The analysis of these studies does not suggest explanations for these inconsistent gender differences.

The results appear to be more consistent if we consider the role played by age.

Advanced age can accentuate or attenuate the effects observed in almost all the cognitive domains considered. Also, some studies [58,65,89] show that lower arterial blood pressure, both diastolic and systolic, in the populations over 75 years of age is correlated with worse cognitive performance, by considering both global cognitive functioning [58], and specific cognitive domains [65]. In the old population, the ageing process may determine a pattern characterised by high systolic blood pressure and low diastolic blood pressure. This pattern appears to be associated with β-amyloid (Aβ) deposition in the brain [103] and with the presence of Alzheimer disease [104]. Thus, atherosclerosis, hypotension and excessive treatment of hypertension may induce cerebral hypoperfusion, ischemia and hypoxia, leading to neurodegenerative processes that speed up the clinical manifestations of cognitive impairment and dementia [105]. Furthermore, given the physiological changes in the brain due to ageing, the elderly would be more vulnerable to episodes of hypotension, often related to brain damage that could explain cognitive impairment [26].

Interestingly, in some studies, diastolic but non-systolic blood pressure was related to cognitive impairment [44,47,53,57]. Diastolic blood pressure affects the small arteries, which undergo progressive vascular atrophy with age. In the presence of high diastolic blood pressure, this atrophy may be responsible for the ischemic lesions of white matter and some age-related cognitive impairments [105,106].

These assumptions were also confirmed by the longitudinal studies, which identified that high blood pressure in adulthood is predictive of poor cognitive performance in old age (i.e., [73,75,76]).

In conclusion, there is substantial evidence about the link between blood pressure and cognitive functioning. The effects of high blood pressure on the functional and structural integrity of the cerebral microcirculation could explain this relationship [107]. Exposure to elevated blood pressure promotes oxidative stress and microvascular lesions [107]. Furthermore, it appears to be related to alterations in the permeability of the encephalic blood barrier [108].

Recent advances in neuroimaging and haemodynamic monitoring have permitted a better understanding of the mechanisms by which high blood pressure affects cognitive functioning. High blood pressure, especially in adulthood, has been identified as a risk factor for cerebral atrophy, white matter lesions, microstructural damages of small cerebral vessels and a decrease in brain metabolism [9,62]. The alterations caused by hypertension to the neurovascular units and the neural biochemical environment make the brain vulnerable and predispose it to the development of dementia, although they are not the exclusive causes of neurodegeneration [29,30].

However, vascular alterations could cause neuroinflammation, micro-haemorrhages and alterations in capillarization of brain tissue (cerebral-microvascular rarefaction) that contribute to changes in cerebral blood flow [109]. These dysfunctions can further alter the neurovascular coupling response, i.e., the ability to self-regulate blood flow based on neural metabolic demand [107]. The consequences of these structural and functional changes, in particular of cerebral blood flow, could be responsible for the cognitive impairment associated with the increase in blood pressure. The apparent absence of symptomatology related to hypertensive condition is associated with an absence of diagnosis and treatment. This aspect could be the cause of cumulative neuroanatomical changes [10] that, in the long period, may result in severe cognitive impairments. From this point of view, it would be interesting a longitudinal evaluation of arterial blood pressure, not necessarily associated with the condition of hypertension and of cognitive functions to assess this relationship between blood pressure trend and cognitive functioning over time.

Some psychopathological symptoms can also mediate the relationship between blood pressure and cognitive functioning. Hypertensive patients often present depression, alexithymia [110], inadequate strategies to cope with stress [111] and poorer health-related quality of life. These psychological conditions could depend on the recognition that they have “a problem” that needs treatment for all of their life [112]. Moreover, depression and reduced quality of life are independently associated with cognitive impairment [112], and this association might well explain the positive relationship between hypertension and cognitive decline.

Another aspect that can be associated with hypertension is sympathetic predominance. Sympathetic predominance seems to precede the rise in blood pressure and the development of hypertension. In fact, the autonomic profile of hypertensive and normotensive people is different [106]. Autonomic dysfunctions, in particular, a sympathetic predominance, seem to be related to cognitive impairment and to worse performance in different cognitive domains (for a review see [113]).

However, despite the analysis of some autonomic parameters, such as Heart Rate Variability, could improve the knowledge of underlying pathophysiology, no specific study has analysed in hypertensive patients the biomarkers of sympathetic and parasympathetic activity by also considering cognitive functioning.

It would also be useful to investigate further aspects that have been chosen to exclude from this review. Among these, the study of the reactivity of blood pressure and the role of drug therapy could be relevant. Some studies (e.g., [114]) have shown that antihypertensive drugs, such as beta-blockers, slow down cognitive decline. Research on the reactivity of arterial blood pressure indicates that higher basal reactivity is associated with worse cognitive performance (for a review see [115]).

In general, this review highlights how some cognitive domains are more investigated than others. While high attention was expressed for executive functions and memory, less interest was shown for attention, language, processing speed and visuospatial skills. These cognitive functions have probably been disregarded because these domains are rarely associated with early alterations in cognitive impairment. However, it would be interesting to broaden the analysis of cognitive functioning by fully involving these domains, as they are those that present more inconsistent results and therefore a more criticism.

Although we have tried to control the research methodology of the analysed studies, this work presents some limitations that could undermine the generalizability of the considerations made. As in all systematic reviews, the first limit is related to the populations considered and to the higher heterogeneity of samples and measurements. The second limit is due to the lack of quantitative analysis carried out through a meta-analysis that would give greater force to the inferences, through the examination of the effects dimension.

Another limit could be the publication bias. The choice to include only academic articles published in peer-review journals may have limited the selection of only those studies that have obtained results in line with the literature. So, the results could have an overestimation of the relationship. Also, the choice to select only the studies published in English could have led to the deletion of studies conducted on other populations. Furthermore, the use of rigid inclusion criteria despite permit better control of phenomenon, it limited the number of studies analysed. However, the main limits are due to the measurement of blood pressure. All studies follow the international guidelines by using an ambulatory blood pressure measurement, which does not allow a detailed analysis of the circadian rhythm of blood pressure. This assessment could lead to classification errors due to an overestimation (e.g., the white coat effect) or an underestimation (e.g., masked hypertension) of blood pressure values. An assessment, including the circadian trend of blood pressure (as the Ambulatory Blood Pressure Monitoring), could be useful in the analysis of the relationship between vascular alterations and cognitive impairment.

## 5. Conclusions

In this systematic review, we tried to investigate the relationship between blood pressure and cognitive functioning by analysing studies selected according to rigid inclusion and exclusion criteria. These conditions allow excluding populations with dementia or severe cardiovascular disorders. The findings of this review underline a substantial relationship between arterial blood pressure and cognitive performance throughout the lifespan. The results show that high blood pressure influences cognitive impairment before a well-established diagnosis of dementia and in the absence of severe cardiovascular diseases such as strokes. Our results showed that this relationship appears to be independent by demographic factors (gender and education), medical co-morbidity (diabetes), and psychiatric disorders (depression), but presents a direct association with age. Opposite patterns based on the groups’ age can be found. Higher systolic or diastolic blood pressure is associated with cognitive impairment in a young adult population (for references see Table 3), while in older individuals this relationship is reversed, generating a sort of “cardiovascular paradox” that allows considering high arterial blood pressure as a protective factor for cognitive health (for references see Table 3).

In conclusion, this review shows that the cardiovascular system and the neurocognitive system do not operate in isolation but are related. Despite some limitation, blood pressure could be considered an early biomarker for the measurement of cognitive impairment in populations without dementia or stroke. The relationship between hypertension and cognitive impairment represents one more reason to emphasise the need for primary prevention in preliminary stages of pre-hypertension aimed at blood pressure control.

## Figures and Tables

**Table 1:** Search scripts.

Issue	Database	Script
Blood Pressure	Pubmed	(cognit * or neuropsychology *) and (blood pressure or hypertens * or high blood pressure)
	PsychINFO	(cognit * or neuropsychology *) and (blood pressure or hypertens * or high blood pressure)

**Table 2:** Characteristics of the selected studies.

Study	Participants						Cognitive Domain							Elevated Blood Pressure	Links to Cognitive Impairment	Follow-Up (Years)
	Group	N	Age M (SD)	Sex (% men) ^a^	SBP M (SD) ^a^	DBP M (SD) ^a^	GC	ME	EF	LG	AT	PS	VS			
Elias et al., 1990 [41]		301	44.1 (12.8)	45.1	135.0 (26.1)	88.0 (15.5)	✓	✓	✓				✓	SBP/DBP	Positive	
Elias et al., 1995 [42]		1695	67.2 (7.5)	59.4	131.1 (17.6)	82.1 (9.4)	✓	✓	✓	✓	✓			SBP/DBP	Not direct	28
Launer et al., 1995 [43]		3735	52.7 (4.7)	100	131.3 (16.6)	82.9 (9.4)	✓							SBP	Positive	28
Cacciatore et al., 1997 [44]		1106	73.9	55	145.3 (19.0)	82.0 (9.2)	✓							DBP	Positive	-
Cerhan et al., 1998 [45]		13,913	45–69	44				✓	✓			✓		SBP/DBP	Positive in women	
van Boxtel et al., 1997 [46]		943	25–80	50.3			✘	✓	✓			✓		SBP/DBP	Non-Linear	-
Kilander et al., 1998 [47]		999		100		82.0 (10.0)	✓		✓				✓	DBP	Positive	20
Swan et al., 1998 [48]		717	76.3 (4.1)		134.2 (8.8)	85.8 (5.9)	✓	✓	✘			✓	✓	SBP	Positive	25–30
Glynn et al., 1999 [49]		3809	>65	38	145.6 (6.2)		✓	✓						SBP	J curve	9
Di Carlo et al., 2000 [50]		3134	74.0 (5.6)				✓							SBP/DBP	No Relationship	
Knopman et al., 2001 [51]		10,882	56.8 (5.7)	44				✓	✓			✓		SBP/DBP	Positive	6
Bohannon, et al., 2002 [52]		3202	73 (6.29)	33	143.1 (20.3)	79.2 (11.8)	✓							SBP	J curve	3
Izquierdo-Porrera and Waldstein, 2002 [53]		43	59.0 (11.2)	17.0	136.0 (21.0)	78.0 (11.0)		✓	✓		✘		✓	DBP	Positive	-
Morris et al., 2002 [54]		5816	64–104	39			✓	✓				✓		SBP/DBP	U curve	
Pandav et al., 2003 [55]		5446	74.1 (5.7)	43	141.3 (18.4)	76.2 (9.9)	✓	✓						Low DBP	Positive in Indian	-
Elias et al., 2003 [56]	Men	551	65.7 (6.9)	100	131.4 (14.6)	82.7 (8.3)	✓	✓	✘		✘		✓	SBP/DBP	Positive only in men	4–6
	Women	872	67.2 (7.3)	0	131.7 (16.9)	80.1 (8.4)										
Reinprecht et al., 2003 [57]		186	68.0					✓		✓			✓	DBP	Positive	13
Kähönen-Väre et al., 2004 [58]		650	>75	26.3	157.9 (2.0)	82.3 (1.0)	✓							Lower DBP	Positive	10
Kuo et al., 2004 [59]		70	72.0 (4.0)	55.7	134.4 (16.6)	-		✘	✓				✘	SBP	Positive	-
Hebert et al., 2004 [60]		4284	74.0 (6.4)	48	139.6 (19.6)	77.3 (11.5)	✓							DBP	U curve	6
Waldstein et al., 2005 [61]		847	70.6 (8.5)	59	138.7 (20.0)	82.0 (10.9)		✓	✓		✓	✓	✓	SBP/DBP	U curve	11
Kuo et al., 2005 [62]		2802	73.6 (5.9)	24.1			✓		✓			✓		SBP	Positive	2
Singh-Manoux & Marmot, 2005 [63]	Man	5838	43.9 (6.0)					✓	✓	✓				SBP/DBP	Positive in men	2
	Woman		44.4 (6.0)													
Wharton et al., 2006 [64]		105	19.2 (1.1)	43	114.1 (12.6)	70.7 (9.3)					✓			SBP/DBP	Positive	-
Axelsson et al., 2008 [65]		97	81	100			✓	✓	✓	✓		✓	✓	SBP/DBP	Negative	-
Knecht et al., 2008 [66]		377	64.0 (6.6)	45.4	144.0 (18.8)	85.0 (10.8)	✓							SBP	Positive	-
Gupta et al., 2008 [67]		85	52.0 (7.5)	69.4	137.4 (27.2)	89.7 (18.3)	✓	✓^b^	✓				✓	SBP/DBP	Positive	-
Obisesan et al., 2008 [68]		5724	>60	42.9	139.8	74.7	✓							SBP/DBP	Positive	-
Knecht et al., 2009 [69]		377	64.0 (6.6)	45.4	144.0 (18.8)		✓							SBP	Positive	-
Gunstad et al., 2009 [70]		99	69.2 (7.5)	60.6				✘		✓	✘		✘	SBP/DBP	Negative	-
Arntzen et al., 2011 [71]	Men	2227	58.8 (9.2)		143.3 (19.3)			✓				✓		SBP	Positive	7
	Women	2806	58.2 (9.7)		141.6 (22.6)											
Yeung and Thornton, 2011 [72]		74	66.2 (8.4)	0	120.4 (17.2)	75.1 (11.1)	✓	✓	✓			✓		SBP/DBP	Positive	-
Goldstein et al., 2013 [73]		1385	73.5 (8.9)	48.7	134.1 (14.3)	74.7 (9.0)	✓	✓	✓	✓	✓	✓		SBP/DBP	Positive	3
Crichton et al., 2014 [74]		972	23–98	41			✓							SBP/DBP	Positive	-
Gottesman et al., 2014 [75]		13,476	57.0 (6.0)					✓	✓				✓	SBP/DBP	Positive	20
Yaffe et al., 2014 [76]		3381	18–30					✓	✓			✓		SBP/DBP	Positive	25
Conway et al., 2015 [77]		319	72	34			✓							SBP/DBP	Positive SBP and Negative DBP	-
Goldstein et al., 2017 [78]		844	74.7	36	140.9	76.2	✓	✘	✓	✘	✓			SBP/DBP	Positive	4
Ferreira et al., 2017 [79]		131	67.7 (5.3)	48.1	136.7 (16.1)	73.6 (9.4)		✓						SBP	Indirect relation	7
HYPERTENSIVE vs. NORMOTENSIVE																
Waldstein et al., 1996 [80]	Young normotensive	26	35.1 (3.8)	100	117.9 (8.0)	74.3 (7.9)		✓	✓			✓	✓	SBP/DBP	Positive in young	-
	Young hypertensive	59	35.15 (3.9)		145.6 (13.5)	97.2 (7.1)										
	Middle-aged normotensive	24	46.6 (4.5)		118.3 (10.2)	75.3 (5.2)										
	Middle-aged hypertensive	64	48.1 (4.7)		146.9 (7.9)	97.8 (7.9)										
Harrington et al., 2000 [81]	Hypertensive	107	76.0 (4.0)	45	164.0 (9.0)	89.0 (7.0)		✓			✓		✓	SBP/DBP	Positive	-
	Normotensive	116	76.0 (4.0)	50	131.0 (10.0)	74.0 (7.0)										
André-Petersson, et al., 2001 [82]	Normotensive	72	68	100				✓^b^	✓	✓		✘	✘	SBP/DBP	Positive	-
	Hypertensive 1	166														
	Hypertensive 2	138														
	Hypertensive 3	88														
André-Petersson et al., 2003 [83]	Normotensive	24	68	100				✓^b^	✓	✘		✓	✓	SBP/DBP	Positive	13
	Hypertensive 1	73														
	Hypertensive 2	46														
	Hypertensive 3	25														
Saxby et al., 2003 [84]	Hypertensive	250	76.4 (4.0)	47	165.0 (8.0)	88.0 (7.0)		✓	✓		✓			SBP/DBP	Positive	-
	Normotensive	256		56	131.0 (11.0)	73.0 (7.0)										
Waldstein and Katzel, 2004 [85]	Normotensive:							✓^b^	✘		✓	✓	✓	SBP/DBP	Positive	-
	Men	30	66.8 (6.7)	100	123.2 (10.2)	71.8 (6.7)										
	Women	26	65.1 (6.6)	0	117.3 (10.7)	67.1 (6.9)										
	Hypertensive															
	Men	31	68.9 (6.6)	100	147.4 (13.7)	80.4 (7.5)										
	Women	11	66.1 (5.6)	0	146.2 (13.5)	81.4 (6.9)										
Brady et al., 2005 [86]	Normal blood pressure:							✘	✘		✓^c^		✘	SBP/DBP	No direct	3
	Normotensive	203	66.0 (7.0)		124.4 (9.4)	78.5 (5.9)										
	Controlled	34	68.6 (6.0)		127.2 (7.9)	77.8 (8.1)										
	Hypertensive:															
	Untreated	75	68.4(7.5)		156.8 (16.1)	89.1(11)										
	Uncontrolled	45	69.5(6.1)		15.2(14.3)	89.0(9.4)										
Waldstein et al., 2005 [87]	Normotensive:	101						✓ ^b^	✓	✓		✓	✘	SBP/DBP	Positive	-
	Normal BP		65.8 (6.5)	61	120.0 (10.6)	69.6 (7.2)										
	High BP		67.0 (6.0)	65	145.5 (7.8)	80.9 (5.4)										
	Hypertensive:															
	Normal BP		68.4 (9.8)	69	132.7 (5.4)	76.5 (7.9)										
	High BP		67.6 (5.0)	72	159.3 (8.8)	84.8 (6.5)										
Hannesdottir et al., 2009 [88]	Normotensive	30	68.3 (8.5)	53.3	127.0 (11.3)	74.0 (10.0)	✓	✓	✓		✘	✓		SBP/DBP	Positive	-
	Treated Hypertension	40	69.3(11.3)	60.0	152.0(19.4)	85.0(11.0)										
	Untreated Hypertension	10	57.6(6.1)	50.0	167.0(16.3)	100.0(6.3)										
Huang et al., 2009 [89]	Hypertension	446	93.6(3.4)	31.1	154.8(17.4)	77.0(6.0)	✘				✓			SBP/DBP	No relation	-
	Normotension	336	93.7(3.4)	34.2	120.3(12.3)	66.9(9.3)										
Yeung and Loken Thornton, 2017 [90]	Hypertensive	71	70.3(6.5)	50	136.3(10.2)	73.3(8.6)	✓	✘	✓			✓		SBP	Positive	-
	Normotensive	62	70.2(6.4)	38	119.3(12.8)	71.2(8.4)										

M = mean; SD = standard deviation; SBP = Systolic Blood Pressure; DBP = Diastolic Blood Pressure; ✘ = domain assessed but not impairment by specific study; ✓ = domain assessed and impairment by specific study; GC = global cognition; ME = memory; LG = language; AT = attention; EF = executive functioning; PS = information processing speed; VS = visuoperceptual skills; ^a^ not reported in all studies; ^b^ Visual memory impairment; ^c^ Visual Attention impairment.

**Table 3:** Neuropsychological tests used for the assessment of cognitive domains in the studies on Blood Pressure.

Cognitive Domain	Task	Study
Global Cognition	Composite Score	[41,42,56,60,66,69,74,75]
	Cognitive Abilities Screening Instrument (CASI)	[43]
	Pfeiffer Short Portable Mental Status Questionnaire (SPMSQ)	[52]
	Montreal Cognitive Assessment (MoCA)	[77]
	Groningen Intelligence Test (GIT)	[46]
	Mini-Mental State Examination (MMSE)	[44,47,48,50,54,55,58,62,65,67,68,72,73,78,88,89,90]
Memory	East Boston Memory Test (EBMT)	[54]
	subtest of Kaplan-Albert battery	[56]
	Composite score	[42,72,73,78,79,80,88]
	Verbal Paired Associates (VPA)	[57,65,82,83]
	Digit Span Forward	[67]
	California Verbal Learning Test (CVLT)	[48,61,72,90]
	Tactile Perception Test-Memory	[41]
	East Boston Memory Test	[49]
	Benton visual retention test (BVRT)	[83]
	Logical memory test	[59,61]
	Word Recall List	[45,51,55,71,75,76,83,86]
Language	Subtest of Kaplan-Albert battery	[56]
	Synonyms	[57,65,82,83]
	Boston Naming Test	[73,78]
	paired association naming test	[42]
	Word Learning Task (WLT)	[46]
Attention	Subtest of Kaplan-Albert battery	[56]
	Digit Forward	[42,53,71,78,80]
	Composite score	[73,80,84]
	Spatial Orienting Task	[64]
	Simple Reaction Time	[81]
Executive Function	Composite score	[41,42,46,48,52,59,62,67,71,72,73,78,80,81,86,87,88,90]
	Subtest of Kaplan-Albert Battery	[56]
	Digit Symbol Substitution Test (DSST)	[62,65,82]
	Verbal Fluency	[45,51,75]
	Trial Making Test–B	[47]
	Stroop Task	[76]
Processing Speed	Useful Field of View	[62]
	Trial making test- parte A	[88]
	Digit Symbol Coding task	[45,51,71,72,90]
	Composite score	[80]
	Letter/Digit Substitution Test	[46]
	Digit Symbol Substitution Test	[48,54,75,76]
Visuospatial Abilities	subtest of Kaplan-Albert battery	[56]
	Composite score	[57,65,71,80,81,82,87]
	Visuospatial Working Memory Matrix	[67]
	Trial Making Test-A	[41,47]
	Visual Reproductions Test	[42,62]
	Clock Drawing Test	[53]
	Figure Coping Test	[86]
	Visual Search Task	[64]
